# Combined Comparative Genomics and Gene Expression Analyses Provide Insights into the Terpene Synthases Inventory in *Trichoderma*

**DOI:** 10.3390/microorganisms8101603

**Published:** 2020-10-18

**Authors:** Isabel Vicente, Riccardo Baroncelli, María Eugenia Morán-Diez, Rodolfo Bernardi, Grazia Puntoni, Rosa Hermosa, Enrique Monte, Giovanni Vannacci, Sabrina Sarrocco

**Affiliations:** 1 Department of Agriculture, Food and Environment, University of Pisa, 56124 Pisa, Italy; rodolfo.bernardi@unipi.it (R.B.); grazia.puntoni@unipi.it (G.P.); giovanni.vannacci@unipi.it (G.V.); sabrina.sarrocco@unipi.it (S.S.); 2 Department of Microbiology and Genetics, Spanish-Portuguese Institute for Agricultural Research (CIALE), University of Salamanca, Campus Villamayor, 37185 Salamanca, Spain; riccardobaroncelli@gmail.com (R.B.); me.morandiez@gmail.com (M.E.M.-D.); rhp@usal.es (R.H.); emv@usal.es (E.M.)

**Keywords:** functional gene differentiation, gene regulation, genomic characterization, terpene synthases, *Trichoderma*

## Abstract

*Trichoderma* is a fungal genus comprising species used as biocontrol agents in crop plant protection and with high value for industry. The beneficial effects of these species are supported by the secondary metabolites they produce. Terpenoid compounds are key players in the interaction of *Trichoderma* spp. with the environment and with their fungal and plant hosts; however, most of the terpene synthase (TS) genes involved in their biosynthesis have yet not been characterized. Here, we combined comparative genomics of TSs of 21 strains belonging to 17 *Trichoderma* spp., and gene expression studies on TSs using *T. gamsii* T6085 as a model. An overview of the diversity within the TS-gene family and the regulation of TS genes is provided. We identified 15 groups of TSs, and the presence of clade-specific enzymes revealed a variety of terpenoid chemotypes evolved to cover different ecological demands. We propose that functional differentiation of gene family members is the driver for the high number of TS genes found in the genomes of *Trichoderma*. Expression studies provide a picture in which different TS genes are regulated in many ways, which is a strong indication of different biological functions.

## 1. Introduction

*Trichoderma* is a genus of ubiquitous fungi, comprising beneficious species used as biocontrol agents (BCAs) in crop plant protection due to their ability to antagonize and mycoparasitize a wide range of phytopathogens [1]. Some strains promote plant growth [2] and are also able to protect plants against pathogens indirectly by inducing the plant defense responses [3]. The beneficial effects of *Trichoderma* spp. are supported and often rely on the secondary metabolites (SMs) they produce [4,5,6], and the biological roles associated to these metabolites has been extensively reviewed [7,8,9,10,11,12]. These fungi produce a wide variety of SMs in a strain-dependent manner [13], with peptaibols, polyketides and terpenes as the most relevant [14].

*Trichoderma* spp. are reported to produce a broad diversity of terpenoids, including volatile compounds [15]. Terpenoids play important roles in the physiology of *Trichoderma* and in the interactions with other organisms, acting as toxins, chemical messengers, structural components of membranes, regulators of genes related to stress, and inducers of plant defense responses [15,16]. Despite their huge variety, all fungal terpenes are synthesized from few precursors by terpene synthase (TSs) enzymes. Isopentenyl–pyrophosphate and its isomer dimethyl-allyl pyrophosphate, both synthetized from acetyl-coA, are the five carbon (C) isoprene building blocks for the biosynthesis of linear polyprenyl pyrophosphates: C10 geranyl pyrophosphate (GPP), C15 farnesyl pyrophosphate (FPP) and C20 geranylgeranyl pyrophosphate (GGPP) [17]. These molecules are synthesized by the isoprenyl pyrophosphate synthases (IPSs) and constitute the precursors that undergo further modifications by terpene cyclases (TCs) and prenyl transferases (PTs), the core enzymes mediating the committed steps in terpenoid biosynthesis [18]. According to the origin of their scaffolds, terpenes can be distinguished in those exclusively formed by isoprenyl units (C10 monoterpenes, C15 sesquiterpenes, C20 diterpenes, C25 sesterterpenes and C30 triterpenes), and those of mixed origin (meroterpenoids, indole terpenoids and indole alkaloids).

Although many terpenes have been isolated from *Trichoderma* species, there is no extensive information about TS genes involved in their biosynthesis, and only a few members of the TS family have been experimentally characterized [19]. Functional characterization of TS genes in *Trichoderma* has been mainly focused on the trichodiene synthase (TRI5)-encoding gene, which catalyzes the first committed step in the biosynthesis of trichothecenes harzianum A and trichodermin in *T. arundinaceum* and *T. brevicompactum*, respectively [20,21,22,23,24,25]. Other *Trichoderma* TS genes experimentally characterized are *erg-20* of *T. reesei*, encoding a farnesyl pyrophosphate synthase [26], and *vir4*, required for the biosynthesis of mono- and sesquiterpenes in *T. virens* [27]. Furthermore, genome mining studies have assessed the complete TS-gene family in *Trichoderma*, however, in those cases, the diversity of the genus has been mainly limited to three species—*T. virens*, *T. atroviride* and *T. reesei* [19], or the study has been merely quantitative [28,29,30].

Given the scarce information available about the diversity within the TS-gene family in *Trichoderma*, the first part of this work was focused on the genomic characterization of the whole TS arsenal of 21 strains belonging to 17 *Trichoderma* spp., thus covering a wide number of species with different lifestyles. This provides the most extensive overview of the terpenoid biosynthetic potential of the genus and reports the distribution of each class of TSs across the species. In addition, aimed at shedding some light on the environmental signals regulating the TS genes in *Trichoderma*, in the second part of the work we assessed the expression patterns of nine TSs in different conditions associated to the ecology of these fungi, using *T. gamsii* T6085 as a model. During the past 10 years, *T. gamsii* T6085 has been evaluated as BCA against *Fusarium graminearum*, the most aggressive causal agent of Fusarium Head Blight (FHB) on wheat. T6085 is able to reduce the growth of the pathogen as well as the production of deoxynivalenol (DON) [31,32], growing in the presence of high DON concentrations (50 ppm) and reducing FHB symptoms and the development of *F. graminearum* perithecia on wheat straw [33,34,35,36]. In addition, the fungus establishes a beneficial interaction with wheat roots, behaving as an endophyte and inducing the plant defense responses [36]. The versatile lifestyle of *T. gamsii* T6085 enabled us to investigate the TSs expression patterns in artificial media simulating stress conditions, as well as during the interaction of the fungus with wheat roots, and during the interaction with *F. graminearum* on wheat spikes, thus furnishing interesting novel results about the regulation of these genes and their role in the ecology of *Trichoderma*.

## 2. Materials and Methods

### 2.1. Genomic Platform

Genomes including gene annotation, available in public databases (National Center for Biotechnology Information [37]; Joint Genome Institute -*JGI*- [38], comprising 21 strains belonging to 17 *Trichoderma* species, were used for computational analyses (Table S1) [39,40,41,42,43,44,45,46,47,48,49]. Model organism *Beauveria bassiana* was used as outgroup due to its close phylogenetic relationship with *Trichoderma* spp.

### 2.2. Prediction of TS Proteins

InterProScan v5.44-79.0 [50] was used to identify TS proteins in *Trichoderma* spp. and *B. bassiana* proteomes, based on the terms associated to their conserved domains (Isoprenoid synthase domain: IPR008949, Terpene cyclase/prenyl transferase domain: IPR008930). Pfam, PIRSF, Prosite, and Panther algorithms included in the interface, were used to identify motifs relatives to sites, superfamily membership, variants of prenyl transferase domains, and trans-membrane (TM) regions associated to TS proteins.

### 2.3. Genomic Characterization of TS Proteins

In order to characterize TS enzymes in *Trichoderma* spp. and *B. bassiana* we used a combination of different approaches: (i) PTs proteins were identified based on Pfam and Panther motifs, which in turn enabled differential identification of TCs proteins; (ii) conserved aspartate-rich metal-binding motifs associated to Class I (D[D/E]xx[D/E]) and Class II (DxDD) TS-folds [51] of mono- and bifunctional enzymes were identified searching for profile Hidden Markov Models (pHMM) in the amino-acidic sequences; (iii) substrate-specificity and putative functions were assigned by phylogenetic analysis: *Trichoderma* spp. and *B. bassiana* TS proteins were aligned by MAFFT v7.450 [52] along with TS proteins of known functions and described in literature (Table S2) [53,54,55,56,57,58,59,60,61,62,63,64,65,66,67,68,69,70,71,72,73,74,75,76,77,78,79]. Phylogenetic tree was built with: MrBayes [80], FasTree v2.1.11 [81] and PhyML v3.3.2 [82]. The best substitution model was obtained using ProtTest [83]. Phylogenetic tree was reconstructed using the WAG + I evolutionary model [84]. The probabilities and bootstrap values threshold were 50%. Phylogenetic trees were visually checked and topology conservation evaluated. Sequences used for alignments and corresponding to each phylogenetic cluster identified were individually screened for conserved domains using InterProScan as described above.

### 2.4. Fungal and Plant Material

*Trichoderma gamsii* T6085 (*Tgam*) [42] and *Fusarium graminearum* ITEM 124 (*Fgra*) [85] were grown on PDA (Sigma-Aldrich, Milano, Italy) plates at 25 °C, 12 h/12 h light/darkness. Seeds from *Triticum aestivum* cv. *Apogee* (wheat) were sown in pots in a potting mix and incubated in a growth chamber with a photoperiod of 16 h light/8 h dark, at 20 °C/22 °C. Before all experiments, wheat seeds were surface sterilized with a NaClO solution (0.6% active chlorine) for 3 min on shaking, followed by three washing steps of 10 min each with sterile distilled water. Seeds were stored in sterile distilled water at 4 °C for 3 days for vernalization.

### 2.5. Liquid Cultures of Tgam

Mycelium of *Tgam* was obtained following a two-step liquid culture procedure. Spores of *Tgam* were collected from 1-week-old PDA plates and inoculated in 50 mL flasks containing 25 mL of minimal medium (MM) 0.9% sucrose (9 g L^−1^ sucrose – Panreac, Milan, Italy-, 1 g L^-1^ NH_4_NO_3_ – Carlo Erba, Milan, Italy-, 5 g L^−1^ C_4_H_12_N_2_O_6_ – J.T. Baker, Milan, Italy-, 1 g L^−1^ K_2_HPO_4_ – Carlo Erba, Milan, Italy-, 0.5 g L^−1^ MgSO_4_ – Carlo Erba, Milan, Italy-, 0.13 g L^−1^ CaCl_2_ – Panreac, Milan, Italy-, 0.1 g L^−1^ NaCl – Carlo Erba, Milan, Italy-, 0.0183 g L^−1^ FeSO_4_ – Carlo Erba, Milan, Italy-, 0.0035 g L^−1^ ZnSO_4_ – J.T. Baker, Milan, Italy-, 0.002 g L^−1^ MnCl_2_ – Carlo Erba, Milan, Italy-) at a final concentration of 10^6^ spores ml^−1^. Flasks were incubated at 28 °C on a rotatory shaker at 180 rpm for 60 h. Mycelium was collected by centrifugation at 10,000 rpm for 10 min and supernatants were discarded. Mycelial pellet was resuspended in sterile distilled water and centrifugated at 10,000 rpm for 10 min to wash it. Mycelium was then inoculated in 50 mL flasks containing 25 mL of MM, MM 0.9% sucrose, and MM modified by adding different stressors, such as 0.5 mM H_2_O_2_ (Panreac, Milan, Italy), only 0.01% of NH_4_NO_3_ and C_4_H_12_N_2_O_6_ (N starvation), and 200 mM NaCl. Flasks were incubated at 28 °C on a rotatory shaker at 180 rpm for 4 days. Mycelium was collected by filtration using Miracloth (475855-1R, Merk, Milan, Italy), frozen in liquid N_2_ and stored at −80 °C until RNA extraction. Three independent biological replicates were included for each condition.

### 2.6. Tgam Interactions in FHB Scenario

Wheat seeds were sown in pots in a potting mix (Esselunga, Pisa, Italy) and incubated in a growth chamber with a photoperiod of 16 h light/8 h dark, at 20 °C/22 °C respectively, until plants reached the anthesis stage (5 weeks). Three biological replicates of three plants each were inoculated per each condition, i.e., *Tgam* alone, *Fgra* alone and *Tgam* + *Fgra* theses. For *Tgam* inoculation, spores were collected by washing 1-week-old PDA plates with 20 mL of sterile 0.01% Tween-80 (Carlo Erba, Milan, Italy) solution, and a 10^7^ spores mL^−1^ suspension was sprayed on spikes of *Tgam* alone and *Tgam* + *Fgra* plants. Plants were covered with a white bag, previously moistened inside with water to maintain humidity, and with a black bag to facilitate penetration by the fungus. Plants were incubated in a growth chamber for 48 h in the same conditions described above. For inoculation of the pathogen, conidia of *Fgra* were collected by washing 2-week-old PDA plates with 20 mL of sterile 0.01% Tween-80 solution, and a 10^5^ spores mL^−1^ suspension was sprayed on spikes of *Fgra* alone and *Tgam* + *Fgra* plants. Plants were covered with a white bag, previously moistened inside with water, and a black bag was placed above to facilitate penetration by the pathogen. Plants were incubated in a growth chamber in the same conditions described above for an additional 24 h. Bags were then removed for plant aeration and after 1 h, white bags were placed back for an additional 24 h. Six days after inoculation of *Fgra*, from three to four spikes colonized by the fungi were collected from each biological replicate (10 spikes in total for each condition), frozen in liquid N_2_ and stored at −80 °C until RNA extraction. Reduction of FHB symptoms was evaluated by calculating the percentage of healthy and symptomatic spikelets (Disease severity—DS) in *Fgra* alone and *Fgra* + *Tgam* plants. Differences on DS values were determined statistically by ANOVA after angular transformation, (*p* value (*p*) ≤ 0.05) using SYSTAT©v.13.2 software.

### 2.7. Tgam—Wheat Roots Interaction

Four wheat seeds were placed on PDA at 1.5 cm distance from the center of the Petri dish. Plates were sealed with tape and incubated for 24 h at 22 °C. After 24 h, 1 cm^2^ agar plug cut out at the border of 1-week-old colony of *Tgam* was placed in the center of the plates containing wheat seedlings, as well as in PDA plates without plants as controls. Plates were sealed with tape and incubated for 3 days in the same conditions described above. Wheat roots colonized by *Tgam* were collected, frozen in liquid N_2_ and stored at −80 °C until RNA extraction. Mycelium from *Tgam* PDA control plates was collected and stored until use. Three independent biological replicates were included for each condition.

### 2.8. RNA Extraction and cDNA Synthesis

Fungal biomass from liquid cultures, wheat roots colonized by *Tgam* and fungal mycelium from the *Tgam*–root interaction control plates were ground in liquid N_2_ using pre-chilled mortar and pestle. Samples containing 100 mg of powder were used for total RNA extraction using the RNeasy^®^ Plant Mini Kit (Qiagen, Milan, Italy), according to the manufacturer’s instructions. Wheat spikes colonized by the fungus were ground in liquid N_2_ using pre-chilled mortar and pestle. Samples containing 300 mg of powder were used for total RNA extraction according to the method described by Logemann et al., 1987 [86]. RNA samples were treated with DNase I (DNase I Amplification Grade, AMPD1 Sigma-Aldrich, Milan, Italy) for gDNA removal, according to the manufacturer’s instructions. A total of 400 ng of RNA were used for cDNA synthesis using Maxima First Strand cDNA synthesis kit (K1642, Thermo Scientific, Milan, Italy) according to the manufacturer’s instructions.

### 2.9. Gene Expression Analyses

Terpene synthases gene expression was analyzed by quantitative real-time PCR performed in Rotor-Gene Q cycler (Qiagen) with QuantiNova SYBER^®^ Green PCR Master Mix 2X (Qiagen) (TS genes used for expression analysis are listed in Table 1). All PCR reactions were performed in triplicate (technical replicates) for each biological replicate in a total volume of 20 µL for 40 cycles under the following conditions: initial activation, 95 °C, 2 min; 40 cycles of denaturation, 95 °C for 5 sec and combined annealing/extension, 60 °C for 10 sec. Threshold cycles (Ct) were calculated with Rotor-Gene Q Series Software 2.3.1 using the β-*tubulin* gene as endogenous control, which was selected due to its expression stability among other housekeeping genes (*actin* and *transcription elongation factor-1* genes). Data were expressed as 2^−ΔΔ*C*t^ [87]. Values from three biological replicates were consistent and used for ANOVA statistical analysis, using SYSTAT©v.13.2 software. Data from liquid cultures of *Tgam* were analysed by ANOVA and Tukey test (*p* ≤ 0.05) using SYSTAT©v.13.2 software. Primers used for assessing expression patterns of *ts1*, *ts3*, *ts4*, *ts5*, *ts6*, *ts7*, *ts9*, *ts11*, *tri5* genes, and those of β-*tubulin* (Table S3) were checked for efficiency and dimmer formation.

## 3. Results

### 3.1. Characterization of TSs Provides an Overview of the Terpenoid Biosynthetic Potential in Trichoderma

**Figure 1:**
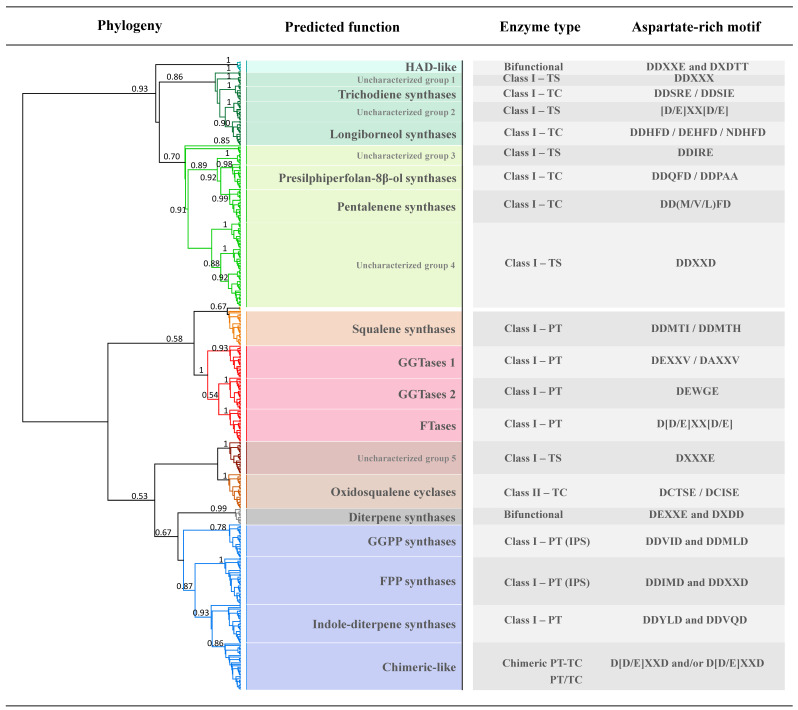
Genomic inventory for terpenoid biosynthesis in *Trichoderma* spp. Terpene synthase (TS) proteins sharing conserved domains are highlighted in different colors: HAD-like (light blue), TRI5 (dark green), terpene synthase C (light green), squalene synthase-phytoene synthase (orange), prenyl transferase (red), squalene/hopene cyclase (light and dark brown), kaurene synthase and/or ent-copalyl diphosphate synthase (grey), and polyprenyl synthase and/or TS C (dark blue). Putative functions of terpene cyclases (TCs) and prenyl transferases (PTs) were assigned based on phylogenetic analysis performed with terpene synthase proteins with known function from filamentous fungi (Table S2) [53,54,55,56,57,58,59,60,61,62,63,64,65,66,67,68,69,70,71,72,73,74,75,76,77,78,79]. Proteins which did not clustered with any known protein were designed as uncharacterized TSs. Aspartate-rich motifs of Class I, Class II and Bifunctional enzymes were identified in the amino-acidic sequences of each group of proteins. Bootstrap values > 50 are shown in the correspondent branches of the tree.

We used an in-silico approach in order to assign putative functions to 387 TS-encoded proteins currently found in the genomes of *Trichoderma* spp., which were found distributed in 15 functional groups. TSs sharing conserved domains and metal-binding motifs clustered in the same phylogenetic group, each one highlighted in a different color (Figure 1). TSs accession numbers, TS-content per each species showing specific portions of the TS inventory, and phylogenetic tree including protein accession numbers are available in Table S4 and Figure S1.

Analysis revealed specific TSs sharing N-terminal HAD-like (Pfam 13419; PTHR43611:SF3) and C-terminal TS domains (IPR008930) (light blue color in Figure 1), which seems to be exclusive of species belonging to the clade Viride (Table S4). Although they did not cluster with known TSs, the presence of both Class I DDxxE and Class II DxDTT motifs indicates they are bifunctional enzymes.

We found a vast group of sesquiterpene synthases (sesquiTSs) belonging to the TRI5 superfamily (Pfam 06330) (dark green color in Figure 1), which was particularly represented in species of Viride clade (Table S4). It contains 7 TRI5 (PIRSF001388), 15 longiborneol synthases, and two groups of proteins that did not cluster with known TSs (uncharacterized group 1 and 2, respectively). The phylogenetic distribution of TRI5 (Table S4) indicates that it is not a monophyletic trait in *Trichoderma*, opening questions about its evolutionary origin. Species of clade Viride were the only lacking longiborneol synthases, however, they are rich in TSs of “uncharacterized groups 1 and 2”, which can only be found in few species outside the clade Viride (Table S4).

The sister clade of the TRI5-superfamily group (light green color in Figure 1) contains Class I proteins sharing a conserved terpene synthase C domain (Pfam 03936), which can be found in sesquiTS and monoterpene synthases (monoTS). It contains two groups of sesquiTS including 16 presilphiperfolan-8β-ol synthases, 22 pentalenene synthases and two groups of proteins that did not cluster with known TSs (uncharacterized groups 3 and 4, respectively) (Table S4). Presilphiperfolan-8β-ol synthases are absent in Viride species, *T. arundinaceum* and *T. atrobrunneum*, whereas pentalenene synthases were found in all the genomes analysed (Table S4). Although most of these share highly conserved metal-binding motifs, some proteins lack the NSD/DTE triad but contain an additional DDxxD motif. This suggests they could actually synthesize sesquiterpenes others than pentalenene. TSs of “uncharacterized group 4” are widely distributed across species, but are particularly represented in *T. virens* and *T. pleuroticola* (Table S4). Differently, proteins of “uncharacterized group 3” seem to be exclusive to species belonging to the Harzianum clade, and their phylogenetic proximity to both groups of sesquiTS suggests this group is also composed by this type of TSs.

We found a large group of PTs, identified as squalene synthases (SQSs) (Pfam 00494; PTHR11626:SF2; PS01044) (orange color in Figure 1), showing the conserved TM helix region of 23 residues in their C-terminal [88], and enzymes involved in protein prenylation [89] (red color in Figure 1), such as type I geranylgeranyl transferases (GGTases 1) (PTHR11774:SF4), type II geranylgeranyl transferases (GGTases 2) (PTHR11774:SF11) and farnesyl transferases (FTases) (PTHR11774:SF6). They are present in single copy in the genomes of *Trichoderma*, but the additional SQS found in *T. pleuroti* indicates that at least one SQS is probably pathway-specific (Table S4).

Oxidosqualene cyclases (OSCs) (Pfam 13249; Pfam 13243; PTHR11764; PS01074) (light-brown color in Figure 1) are present in single copy in the genomes of *Trichoderma*, showing DCTSE or DCISE aspartate-rich motifs, both variants of the classical DCTAE reported in these proteins [75], and five conserved QW motifs responsible of strengthening the structure of the enzyme [90].

TSs from the sister clade of OSCs are in single copy in all the genomes and contain a conserved squalene synthase-phytoene synthase domain (Pfam 00494; PTHR21181:SF13) (dark brown color in Figure 1), but they did not cluster with SQSs and neither with reference lycopene-phytoene synthases (uncharacterized group 5).

Copalyl-pyrophosphate/Ent-kaurene synthases (CPS/KS) (PTHR31739:SF4; PIRSF 026498) (grey color in Figure 1) were found in *T. asperellum*, which known for its ability for gibberellin biosynthesis [91]. CPS/KS clustered with other Class II bifunctional enzymes of species from clades Longibrachiatum and Brevicompactum (PTHR31739:SF4), but the low sequence similarity of these with CPS/KS indicates they are diterpene synthases (diTS) not involved in ent-kaurene biosynthesis.

The last cluster (dark blue color in Figure 1) contains proteins sharing a conserved polyprenyl synthase domain (Pfam 00348). Within this group, GGPP synthases (PTHR12001:SF47; PS00723; PS00444) and FPP synthases (PTHR11525:SF0; PS00723; PS00444) were identified, showing the two characteristic DDxxD motifs usually found on these enzymes [51,92]. Some species belonging to Harzianum and Brevicompactum clades have two to three copies of these PTs class, suggesting that at least some of them could be actually pathway-specific (Table S4). Analysis also revealed a set of highly conserved indole diTS, whose biosynthetic products have not been reported yet in *Trichoderma*. The last group contains Class I TSs clustering with known chimeric TSs from fungi (chimeric-like), which were absent in species of clade Viride. Most of them contain only polyprenyl synthase or TS C domains. Nevertheless, we found one protein in *T. asperellum* TR456 containing both domains, which is highly similar to ophiobolin F synthase from *Aspergillus clavatus*, suggesting this specie is able to produce sesterterpenes.

### 3.2. Assessment of the Genomic Context of tri5 Genes Reveals Its Potential Involvement in Unknown Biosynthetic Pathways in Trichoderma

**Figure 2:**
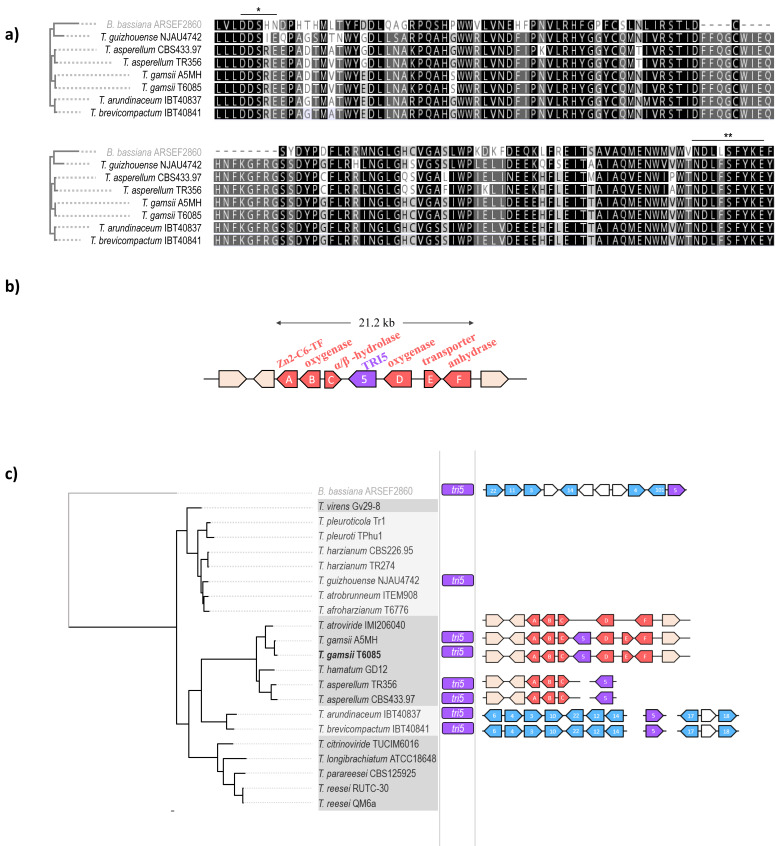
Trichodiene synthases in *Trichoderma* spp. (**a**) Phylogenetic relations among trichodiene synthase (TRI5) proteins in *Trichoderma* spp. and multiple alignment of the active center of the proteins were obtained using MAFFT v7.450 [52] and FasTree v2.1.11 [81]. Aspartate-rich metal binding motifs are indicated with asterisks. (**b**) Biosynthetic gene cluster containing *tri5* and putative functions of the enclosing genes found in strains of *T. gamsii*. (**c**) Phylogenetic distribution of *tri5* and their associated biosynthetic gene clusters in *Trichoderma* spp. Phylogenetic relations of *Trichoderma* spp. were obtained using MAFFT v7.450 [52] and FasTree v2.1.11 [81], using concatenated alignment of *actin*, *calmodulin* and *transcription elongation factor-1* genes. Phylogenetic clades, according to Kubicek et al. (2019) [30], are shown in two different grey colors. Genes belonging to variants of the TRI loci reported in species of the clade Brevicompactum and *B. bassiana*, respectively, are shown in light blue. Genes found associated to *tri5* (in purple) in *T. gamsii* are shown in red.

The presence of *tri5* orthologs in *Trichoderma* spp. that have not been described as trichothecene-producers, such as *T. gamsii*, *T. asperellum* and *T. guizhouense*, suggests this gene could be involved in the biosynthesis of different trichodiene derivatives. Multi-sequence alignment of TRI5 proteins showed the active center is highly conserved, sharing DDSRE/DDSIE aspartate-rich motif and NDLFSFYKE triad (Figure 2a). 

Pairwise alignments of each TRI5 protein with that of *T. arundinaceum* and *T. brevicompactum* showed 77% of amino-acid identity in *T. guizhouense*, 80–82% in *T. asperellum*, respectively, and 87% in *T. gamsii*.

Assessment of the genomic context of *tri5* genes by antiSMASH 5.0 [93] revealed this gene is included in a 21.2 kb cluster in *T. gamsii*, enclosing another six genes that were named as *A*, *B*, *C*, *D*, *E,* and *F* (Figure 2b). Characterization based on conserved domains and similarity with characterized proteins in other systems enabled the identification of one regulatory protein, Zn2-C6 transcription factor (TF) (*A*); four tailoring enzymes: oxygenase (*B*), alpha-beta hydrolase (*C*), oxygenase (*D*), and carbonic anhydrase (*F*); and one efflux transporter, Major Facilitator Superfamily (MFS) transporter (*E*). 

Alignment of these proteins with the TRI (trichothecene) proteins functionally associated to *tri5* in the trichothecene-producer species of the Brevicompactum clade showed no sequence similarity. Furthermore, the genome of *T. gamsii* lacks the entire set of genes encoding the TRI proteins, with the exception of a distant related homolog of the gene *tri101*, which has already been reported in other *Trichoderma* species [43].

In order to search for homologous proteins in the *Trichoderma* genomes here explored, we used the protein sequences encoded in the cluster found in *T. gamsii* as queries in BLASTp analyses. Genes *A*, *B* and *C* were also found in all the other *Trichoderma* spp. belonging to the Viride clade, with conserved synteny (Figure 2c), and preliminary BLAST analyses suggest that these genes may be originated by horizontal gene transfer (HGT) from a donor belonging to the Eurotiomycetes. In any case, further analyses are needed in order to better understand the evolutionary origins of these genes. Instead, genes *D* and *F* are present in some of the genomes analyzed in closely-related species; while gene *E* seems to be specific to *T. gamsii*.

These findings suggest the origin of a novel *tri5*-associated cluster in *T. gamsii*, which is likely involved in the biosynthesis of trichodiene derivates with unknown functions. According to this, *tri5* could participate in two different sesquiterpene biosynthetic pathways in *Trichoderma*.

### 3.3. Functional Differentiation of TS Family Members as the Driver for the High Genomic Potential for Terpenoid Biosynthesis in Trichoderma

We identified 16 TS-encoding genes in the genome of *Tgam* (Table 1). For gene expression studies, we focused on nine genes encoding Class I TSs—*ts1*, *ts3*, *ts4*, *ts5*, *ts6*, *ts7*, *ts9*, *ts11,* and *tri5*—which represented a highly diverse functional group, well distributed across *Trichoderma* spp., according to our analyses. Thus, we excluded genes encoding Class II proteins and those involved in the biosynthesis of terpene precursors and protein prenylation.

**Figure 3:**
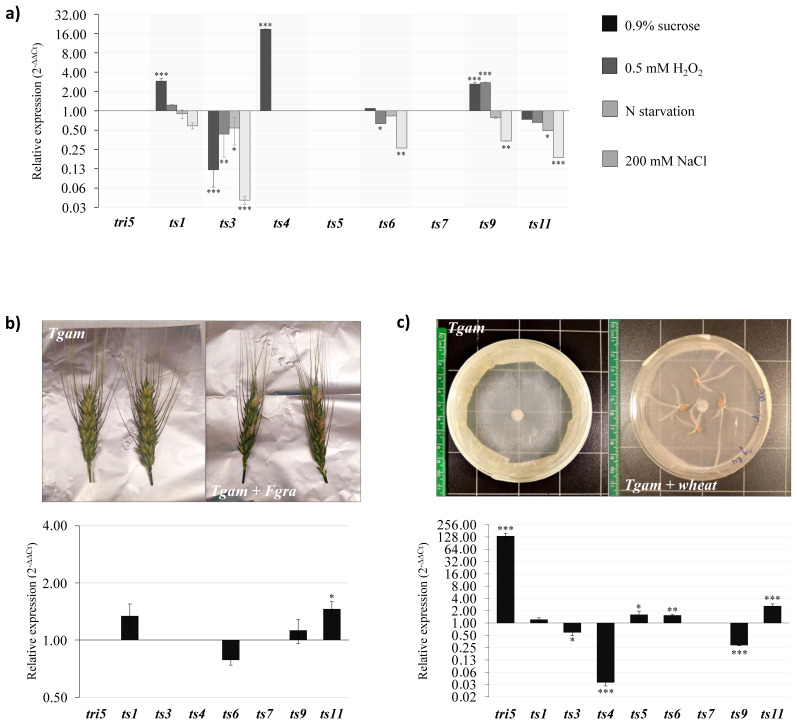
Gene expression studies of terpene synthase genes in *T. gamsii* T6085 in different environment conditions. (**a**) Liquid cultures of *T. gamsii* T6085 (*Tgam*) in different substrates. Total RNA was extracted from 4-day-old mycelium of *Tgam* grown on minimal medium without sucrose (MM) (basal condition, 2^−ΔΔ*C*t^ = 1), or MM with 0.9% sucrose, MM 0.5 mM H_2_O_2_, MM with only 0.01% of nitrogen (N starvation), or 200 mM NaCl, respectively. Grey color bars represent relative expression values of TS genes on each condition. (**b**) Interaction of *Tgam* with *F. graminearum* (*Fgra*) on wheat spikes. Total RNA was extracted from wheat spikes colonized by *Tgam* alone (basal condition, 2^−ΔΔ*C*t^ = 1) or by *Tgam* + *Fgra*, 6 days after inoculation of the pathogen. (**c**) Interaction of *Tgam* with wheat roots. Total RNA was extracted from mycelium of *Tgam* grown on PDA for 3 days (basal condition, 2^−ΔΔ*C*t^ = 1) and from wheat roots colonized by *Tgam* for 3 days. The β-*tubulin* gene was used as control for data normalization. Values are means of three independent biological replicates with the corresponding standard deviation. Fold change in sample relative to control is expressed as 2^−ΔΔ*C*t^. Statistically significant values are indicated with asterisks (*p* ≥ 0.05 no significative; 0.05 > *p* ≥ 0.01 = *; 0.01> *p* ≥ 0.001 = **; *p* < 0.001 = ***).

We firstly investigated changes on TSs expression in 4-day-old liquid cultures of the fungus grown in modified MM (Figure 3a). The availability of C source had contrasting effects on TS gene expression. The addition of 0.9% sucrose did not change the transcript levels of *ts6* and *ts11*. Nevertheless, *ts3* was significantly down-regulated (0.1-fold), whereas an up-regulation was observed in *ts1*, *ts9* and particularly, in *ts4* (18.7-fold). In the same way, the addition of 0.5 mM H_2_O_2_ induced the opposite changes on gene expression, indicating that regulation of TS genes occurs in different manners in response to oxidative stress. While the expression of *ts1* and *ts11* did not change, *ts3* and *ts6* were down-regulated (0.3-, 0.6-fold, respectively). In contrast, *ts9*, encoding a putative indole diTS, was up-regulated (2.7-fold), suggesting that biosynthesis of indole diterpenes might occur in response to oxidative stress in *Tgam*. We observed that N starvation tended to negatively regulate TS genes, although significant differences were only found in *ts3* and *ts11* (0.4-fold). Similarly, the addition of 200 mM NaCl down-regulated the expression of TS genes (0.02–0.33-fold), with the exception of *ts1*, whose expression was not affected. We did not detect transcripts of *tri5*, *ts5* or *ts7* in any of the conditions tested.

Further, we investigated whether a differential expression of TS genes of *Tgam* takes place in the presence of *Fgra* on wheat spikes, where a reduction of FHB symptoms was observed (57.9 ± 4.7% DS) compared to *Fgra* alone plants (88.3 ± 1.2% DS) (Figure 3b). Analysis revealed that *ts1*, *ts6*, *ts9,* and *ts11* were active when *Tgam* was on wheat spikes, regardless of the presence/absence of the pathogen. Expression of *ts1*, *ts6* and *ts9* did not change significantly between the two conditions, indicating that these genes are not particularly involved in this triple interaction. Instead, *ts11* was slightly up-regulated (1.45-fold) when *Tgam* was on spikes with *Fgra*, suggesting the presence of the pathogen could directly induce changes in its expression or could mediate physiological changes in spikes that promoted changes on *ts11* expression. These results indicate that *Tgam* did not induce prominent changes in terpene biosynthesis when interacting with *Fgra* on wheat spikes, under the conditions tested.

Finally, we observed that root colonization affected TSs expression in *Tgam*, which differentially regulated most of its TS genes, with the exception of *ts7*, whose transcripts were not detected in any condition, and *ts1*, in which expression did not change (Figure 3c). This clearly indicates a reprogramming in terpene biosynthesis in *Tgam* when colonizing wheat roots. In particular, a modulation in sesquiterpene biosynthesis occurred in *Tgam* during root colonization. Whereas *ts5* was slightly up-regulated (1.58-fold), the contact with the roots strongly repressed the expression of *ts4* (0.03-fold) and to a lesser extent that of *ts3* (0.59-fold). Nevertheless, the most remarkable difference was found in the expression of *tri5*, which was 134-fold up-regulated when the fungus was on the roots, suggesting that signals from the roots are responsible for triggering the expression of this gene in *Tgam*. In addition, the interaction with the roots up-regulated *ts6* (1.52-fold) and *ts11* (2.58-fold). Since *ts6* was predicted to encode an SQS, this suggests that triterpene biosynthesis was slightly enhanced in *Tgam* when colonizing the wheat roots. Interestingly, *ts9* was found highly down-regulated (0.28-fold), thus suggesting a repression of indole diterpene biosynthesis in the fungus during the interaction with wheat roots.

## 4. Discussion

The impressive number of TSs we found in the genomes analyzed demonstrate that terpenoid biosynthesis has a great impact on the diversity and complexity of SMs in *Trichoderma*. Indeed, the TS-gene inventory of these species (15–23 genes per genome) clearly outnumbers those found in other fungi considered as rich producers of SMs, such as *Aspergillus* spp. (2–10 genes per genome) [30,94]. This reflects the importance of TSs and terpenoids in the ecology of *Trichoderma*. However, most of the *Trichoderma* TSs have not yet been characterized [30], and available information about the diversity of TSs in *Trichoderma* is scarce. Thus, we focused on characterizing the TS-gene arsenal of this genus, providing a complete overview of the nature and diversity of the *Trichoderma* terpenoid biosynthetic inventory.

Although TS family size is very homogeneous within *Trichoderma*, we were able to identify clade-specific TSs, which reflect particular portions of the terpenoid inventory shared by phylogenetically close species. Thus, despite their similar terpenoid biosynthetic potential, the species of *Trichoderma* have adapted their terpene production according to different environmental demands. Species of Viride clade constitute an example, as they are missing in some groups of TSs that are present in the other clades, although they have evolved specific TSs, which are absent in other species of the genus.

According to our results, *Trichoderma* spp. have a huge potential for sesquiterpene biosynthesis. We identified eight groups of sesquiTSs, which constitute almost a third of the total number of TSs found in this work. Species of Viride are particularly rich in sesquiTS belonging to the TRI5-superfamily, and they also contain HAD-like TSs, which are absent in species of other clades. These HAD-like proteins harbor a DxDTT motif, which is a variant of the DxDD found in Class II diTS [95,96]. Shinohara et al. [97] reported that some sesquiTS can contain HAD-like domains and DxDTT motifs, leading to FPP cyclization through a protonation step, instead of by an ionization step. We hypothesized that HAD-like TSs might be particular bifunctional sesquiTS, synthesizing specific metabolites of Viride clade.

Previous studies reported that some *Trichoderma* spp. have the potential to synthesize longiborneol [19], an intermediate of the culmorin biosynthetic pathway [19,56]. According to our results, longiborneol biosynthesis is very widespread in *Trichoderma*, but is absent in species of the Viride clade. Since culmorin production has not been reported in *Trichoderma*, longiborneol could be synthesized as a sole compound or as an intermediate of unknown biosynthetic pathways in these species. Most of these proteins show a conserved D(D/E)HFD motif, which is partially conserved (NDHFD) in proteins of *T. arundinaceum* and *T. brevicompactum*. Site-directed mutagenesis and crystallography studies on TSs have revealed that the first aspartate residue (D) of the metal-binding motif interacts directly with Mg^2+^ [98], and its replacement can lead to anomalous cyclization products or a product mixture [99]. According to this, longiborneol synthases of Brevicompactum species could be actually involved in terpenoid blend formation or in the biosynthesis of newly terpenoids.

We found that most *Trichoderma* spp. can potentially produce presilphiperfolan-8β-ol. This compound is thought to play a central role in the biosynthesis of a wide range of polycyclic sesquiterpenes in fungi [73]. Thus, these TSs may contribute to generating a variety of structurally complex terpenoids in *Trichoderma*. Proteins sharing similarity with fungal pentalenene synthases were also identified, which are involved in the biosynthesis of the parent hydrocarbon of the pentalenolactone family of fungal antibiotics [100]. Nevertheless, the variability found on the structure of the active center of these proteins suggests that some of them probably synthesize sesquiterpenoids others than pentalenene.

Our analyses revealed that single-copy SQS and OSC enzymes provide the linear and cyclic precursors required for triterpene biosynthesis in most *Trichoderma* species. However, the presence of additional SQSs indicates that pathway-specific SQSs could be present as well. In the same way, additional GGPP and FPP synthases may act as donors of terpenoid precursors in specific biosynthetic pathways in members of the clades Harzianum and Brevicompactum. This most likely reflects specific portions of the terpenoid inventory of these species and guarantees an efficient distribution of terpenoid precursors between primary and secondary metabolism.

Harziandione was the first diterpene isolated from *Trichoderma* spp. [101], and a number of these compounds have been recently reported in these species [102,103,104,105,106]. Nevertheless, the low number of diTS found here indicates that the ability of *Trichoderma* spp. for diterpene biosynthesis is scarce and not very widespread within the genus.

Interestingly, our analyses revealed that *Trichoderma* spp. have the potential to produce sesterterpenes and indole diterpenes. Sesterterpenes are rare among terpenoids, and their antimicrobial and nematocidal properties [107] could confer competitive advantages to some *Trichoderma* spp. On the other hand, the production of indole diterpenes has been reported in some Sordariomycetes, being involved in protecting their reproductive structures from fungivores [108]. Many of the indole diterpene-producer fungi establish symbiotic relations with plants, thus, biosynthesis of these compounds may confer ecological advantages on *Trichoderma*–host associations as well [109].

No monoTS were found among the genomes analysed in this work, although production of monoterpenes has been reported in *T. virens* [27,110]. No *bona fide* monoTS have been identified in fungi [111], and the scarce availability of sequences of these enzymes could have led to miss-predict them. Since fungal sesquiTS are able to cyclize GPP [112], we hypothesize that monoTSs of *Trichoderma* could be actually included within “uncharacterized group 4”, since they are phylogenetically close to sesquiTS, and other uncharacterized proteins were found restricted to some clades not including *T. virens* or fell into the TRI5 superfamily.

We found highly conserved *tri5* orthologs in non-trichothecene-producer *Trichoderma* species missing on the entire TRI cluster, some of which have been previously reported [113]. This leaves open some questions about the role of *tri5* in beneficious *Trichoderma* spp., a non-producer of trichothecenes. Unlike other *tri5*-containing species, *tri5* is embedded in a BGC in *T. gamsii*, enclosing tailoring enzymes, a TF and a transporter. The presence of a TF within the cluster suggests a pathway-specific regulation, while the presence of a transporter suggests the production of a sesquiterpenoid with extra-cellular functions. Some of these genes were likely transferred to *Trichoderma* spp. by HGT from Eurotiomycetes, but the entire cluster is only present in *T. gamsii*, suggesting the origin of a novel *tri5*-associated BGC in this species. This cluster could lead to an uncharted trichodiene-derived sesquiterpenic biosynthetic pathway, producing novel metabolites with potential agronomic interest. Since *tri5* seems to be functionally associated to two different BGCs (TRI and that found in *T. gamsii*), we hypothesize this sesquiTS is involved in different metabolic pathways in *Trichoderma*.

The striking genomic potential for terpenoid production of *Trichoderma* spp. found in this work suggests that functional differentiation of gene family members is the driver for the high TS gene numbers of these species. Here we provide a picture showing that different TS genes are differentially regulated, a strong indication of different biological functions.

Availability of C source had contrasting effects on the expression of TS genes, as observed in SMs genes from other fungi [57,114]. Similarly, TS genes were regulated in opposite ways in response to oxidative stress. Association of oxidative stress with SMs biosynthesis in fungi has been extensively demonstrated, and it has been suggested that it is induced to prevent fungi from ROS damage [115]. In particular, up-regulation of *ts9* suggests that biosynthesis of indole diterpenes might occur in *T. gamsii* T6085 under oxidative stress conditions, as it has been observed in *Aspergillus* spp. [116]. Interestingly, the addition of 0.9% sucrose or 0.5 mM H_2_O_2_ to the medium did not induce *tri5* expression in *T. gamsii* T6085, unlike what has been observed in *T. brevicompactum* when grown in the presence of 1% or 2% sucrose or in the presence of 0.5 mM H_2_O_2_ [24]. This suggests different types of regulation of *tri5* in *T. gamsii* T6085 and *T. brevicompactum*.

Nitrogen availability and saline stress have a considerable impact on fungal secondary metabolism [117,118,119]. N starvation and saline stresses negatively regulated TS genes in *T. gamsii* T6085, suggesting terpenoid biosynthesis does not confer particular advantages to the fungus to overcome these stresses.

In *T. arundinaceum*, *tri* gene expression is affected when grown in dual cultures with *B. cinerea*, while polyketides and harzianum A (HA) produced by the first induce changes in some *B. cinerea* genes linked to its virulence [23]. Since *T. gamsii* T6085 is able to suppress *F. graminearum* on wheat spikes and to reduce DON production by the pathogen [31,32,34,35], we investigated whether a differential expression of TS genes of *T. gamsii* T6085 occurs when the fungus interacts with *F. graminearum* on wheat spikes. However, our results show that the presence of the pathogen did not induce prominent changes in TS expression in *T. gamsii* T6085 when both fungi were on wheat spikes, although *ts11* was found slightly up-regulated. Gene expression patterns are highly dynamic, and more extensive time-course experiments are needed to provide more information about the role of TSs in this scenario.

Root colonization by *Trichoderma* is an intimate relationship involving a tightly regulated exchange of molecular signals including SMs [120]. When *Trichoderma* colonizes the roots, it releases a variety of SMs that promote substantial changes in plant biochemistry, which in turn, cause changes in the fungal physiology [8]. Our results indicate that a significative reprogramming of terpene biosynthesis occurs in *T. gamsii* T6085 when it colonizes wheat roots. Down-regulation of *ts9* suggests that root colonization induces a repression in indole diterpene biosynthesis in the fungus. In addition, the contrasting effects observed in the expression of sesquiTS and the up-regulation of the SQS suggest that root colonization induces a modulation on sesquiterpene and triterpene biosynthesis in *T. gamsii* T6085 through FPP as the central node. The absence of *tri5* expression in response to wheat spikes and the strong up-regulation observed during root colonization was remarkable, as it suggests that signals from the roots induce the expression of this gene in *T. gamsii* T6085. Although activation of *tri5* usually leads to the production of phytotoxic compounds, such as trichodermin in *T. brevicompactum* [24], HA produced by *T. arundinaceum* lacks phytotoxic activity and has a crucial role in plant protection against *B. cinerea* [21]. This example illustrates how different ecological demands led to an adjustment in metabolic pathways governed by a single gene signature in species within the same fungal genus [28]. In this context, we can imagine the involvement of *tri5* in the biosynthesis of a sesquiterpenoid related to the promotion of beneficial relationships between *T. gamsii* T6085 and wheat plants, a question that must be further addressed by using *tri5*-deletion mutants of the fungus. Results strongly indicate the involvement of TS genes in the interaction of *T. gamsii* T6085 with plant roots, and further studies determining their impact on the plant physiology will provide further information about the roles of these genes in the *Trichoderma*–plant interaction.

## Figures and Tables

**Table 1:** Terpene synthase genes found in the genome of *T. gamsii* T6085. *JGI* accession numbers of each protein are shown in the first column. Protein names are shown in the second column. Putative protein functions derived from computational analysis are shown in the third column. Terpene synthases used in gene expression analyzed are shown in bold.

*JGI* Id.	Name	Putative function
Trigam1|5596	TC4	bifunctional HAD-like
Trigam1|4742	**TRI5**	**trichodiene synthase**
Trigam1|162	**TS5**	**pentalenene synthase**
Trigam1|9843	**TS7**	**sesquiTS**
Trigam1|1824	**TS4**	**sesquiTS**
Trigam1|4947	**TS3**	**sesquiTS**
Trigam1|5367	**TS1**	**uncharacterized 4**
Trigam1|340	**TS6**	**squalene synthase**
Trigam1|3208	TC3	GGTase I
Trigam1|5139	TC5	GGTase II
Trigam1|3927	TC1	FPP transferase
Trigam1|4065	TS8	GGPP synthase
Trigam1|2917	TS10	FPP synthase
Trigam1|9898	**TS9**	**indole diTS**
Trigam1|8345	TC2	oxidosqualene cyclase
Trigam1|6072	**TS11**	**uncharacterized 5**

## Supplementary Materials

The following are available online at https://www.mdpi.com/2076-2607/8/10/1603/s1, Figure S1: Phylogenetics of terpene synthase proteins in *Trichoderma* spp., Table S1: *Trichoderma* spp. and *Beauveria bassiana* genomic platform, Table S2: Terpene synthase proteins functionally characterized in filamentous fungi, Table S3: Primer sequences used for gene expression analysis (5′to 3′), Table S4: TS-gene content per each *Trichoderma* spp.
