# Supplementary material for: Combined Comparative Genomics and Gene Expression Analyses Provide Insights into the Terpene Synthases Inventory in Trichoderma
Source: Microorganisms. 2020 Oct 18;8(10):1603. doi: 10.3390/microorganisms8101603 (PMC7603203; doi:10.3390/microorganisms8101603)

Figure S1. Phylogenetics of terpene synthase proteins in *Trichoderma* spp. Terpene synthases sharing conserved domains are highlighted in different colours and are associated to their respective *JGI* or NCBI accession numbers. HAD-like (light blue), TRI5 (dark green), terpene synthase C (light green), squalene synthase-phytoene synthase (orange), prenyl transferase (red), squalene/hopene cyclase (light and dark brown), kaurene synthase and/or ent-copalyl diphosphate synthase (grey), polyprenyl synthase and/or terpene synthase C (dark blue). Putative functions of terpene cyclases (TCs) and prenyl transferases (PTs) were assigned based on phylogenetic analysis performed with terpene synthase proteins with known function from filamentous fungi (Table S2). Proteins which did not clustered with any known protein were designed as Uncharacterized terpene synthases. Phytoene-lycopene synthases from fungi which did not cluster with any *Trichoderma* protein are shown unboxed. Bootstrap values > 50 are shown in the correspondent branches of the tree.

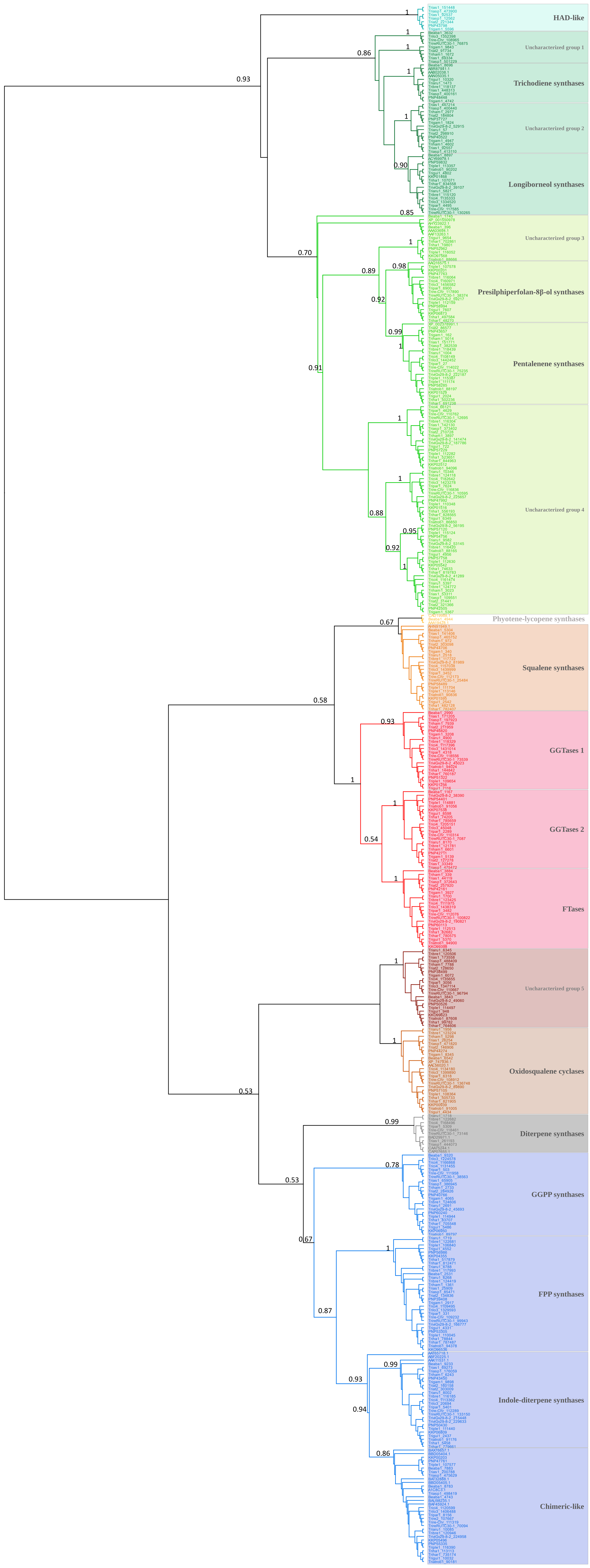

Supplement: Supplementary file 1 [file microorganisms-08-01603-s001.zip › microorganisms-945438_Supplementary/Figure S1.pdf]
