# Supplementary material for: Combined Comparative Genomics and Gene Expression Analyses Provide Insights into the Terpene Synthases Inventory in Trichoderma
Source: Microorganisms. 2020 Oct 18;8(10):1603. doi: 10.3390/microorganisms8101603 (PMC7603203; doi:10.3390/microorganisms8101603)
Supplement: Supplementary file 1 [file microorganisms-08-01603-s001.zip › microorganisms-945438_Supplementary/Table S1.pdf]

Table S1. *Trichoderma* spp. and *Beauveria bassiana* genomic platform. Complete genome sequences of *Trichoderma* spp. and *B. bassiana* were obtained from public databases of NCBI and *JGI* databases. Whole Genome Sequencing (WGS) Project accessions of each species are listed in the second column. Phylogeny according to Kubicek et al. (2019) [30] and information about the isolation of each strain are also shown.

Strain	WGS Project	Phylogeny	Isolate information	Reference
Beauveria bassiana ARSEF 2860	ADAH00000000.1	Outgroup	Isolated from aphid cadavers (Idaho, USA)	[39]
Trichoderma asperellum CBS 433.97	MBGH00000000.1	Clade Viride	Isolated from soil (Maryland, USA)	[40]
Trichoderma asperellum TR356 v1.0	Unreleased	Clade Viride	Isolated from soil (Brazil)	[30]
Trichoderma hamatum GD12	ANCB00000000.2	Clade Viride	Isolated from potato field (UK)	[41]
Trichoderma gamsii A5MH	MTYH00000000.1	Clade Viride	Isolated from wheat cropping soil (Australia)	Unpublished
Trichoderma gamsii T6085	JPDN00000000.2	Clade Viride	Isolated from soil (Ukraine)	[42]
Trichoderma atroviride IMI 206040	ABDG00000000.2	Clade Viride	Isolated from a plum tree (Sweden)	[29]
Trichoderma arundinaceum IBT 40837	PXOA00000000.1	Clade Brevicompactum	Isolated from soil (Iran)	[43]
Trichoderma brevicompactum IBT 40841	PXNZ00000000.1	Clade Brevicompactum	Isolated from soil (Iran)	[43]
Trichoderma citrinoviride TUCIM 6016	MBDI00000000.1	Clade Longibrachiatum	Unknown	[40]
Trichoderma longibrachiatum ATCC 18648	MBDJ00000000.1	Clade Longibrachiatum	Isolated from mud (Ohio, USA)	[40]
Trichoderma parareesei CBS 125925	LFMI00000000.1	Clade Longibrachiatum	Isolated from subtropical rain forest (Argentina)	[44]
Trichoderma reesei QM6a	AAIL00000000.2	Clade Longibrachiatum	Isolated from Salomon Islands	[45]
Trichoderma reesei RUT C-30	JABP00000000.1	Clade Longibrachiatum	Obtained from three rounds of random mutagenesis of QM6a strain	[46]
Trichoderma virens Gv29-8	ABDF00000000.2	Clade Virens	Isolated from rhizosphere	[29]
Trichoderma pleuroticola Tr1	MTYI00000000.1	Clade Harzianum	Submitted as *T. harzianum* - Isolated from carbendazim contaminated vineyard soil (China)	Unpublished
Trichoderma pleuroti TPhu1	MDJU00000000.1	Clade Harzianum	Isolated from oyster mushroom (Hungary)	[47]
Trichoderma harzianum CBS 226.95	MBGI00000000.1	Clade Harzianum	Isolated from soil (England, UK)	[40]
Trichoderma harzianum TR274	NQLC00000000.1	Clade Harzianum	Isolated from soil (Brazil)	[30]
Trichoderma guizhouense NJAU 4742	LVVK00000000.1	Clade Harzianum	Isolated from soil with compost of traditional Chinese medicine (China)	[40]
Trichoderma afroharzianum T6776	JOKZ00000000.1	Clade Harzianum	Submitted as *T. harzianum* - Isolated from soil (Pisa, Italy)	[48]
Trichoderma atrobrunneum ITEM 908	PNRQ00000000.1	Clade Harzianum	Isolated from *Olea europaea* (Puglia, Italy)	[49]
