# Supplementary material for: Combined Comparative Genomics and Gene Expression Analyses Provide Insights into the Terpene Synthases Inventory in Trichoderma
Source: Microorganisms. 2020 Oct 18;8(10):1603. doi: 10.3390/microorganisms8101603 (PMC7603203; doi:10.3390/microorganisms8101603)
Supplement: Supplementary file 1 [file microorganisms-08-01603-s001.zip › microorganisms-945438_Supplementary/Table S2.pdf]

Table S2. Terpene synthase proteins functionally characterized in filamentous fungi.

Strain	Accession	Function	Classification	Reference
Fusarium fujikuroi m567	CAA75244.1	copalyl pyrophosphate synthase/ent-kaurene synthase	Bifunctional Class II Cyclase - diTS	[53]
Fusarium fujikuroi m567	CAD19988.1	phytoene synthase-lycopene cyclase polypeptide	Class I PT GFPP synthase - Class I Cyclase - tetraTS	[54]
Fusarium sporotrichioides NRRL 3299	AAN05035.1	trichodiene synthase	Class I Cyclase - sesquiTS	[55]
Fusarium graminearum GZ3639	ACY69978.1	longiborneol synthase	Class I Cyclase - sesquiTS	[56]
Fusarium graminearum H3	ABR87941.1	trichodiene synthase	Class I Cyclase - sesquiTS	[57]
Fusarium sambucinum R-6380	AAB02038.1	trichodiene synthase *tox5*	Class I Cyclase - sesquiTS	[58]
Sphaceloma maniticola Lu949	CAP07655.1	copalyl pyrophosphate synthase/ent-kaurene synthase	Bifunctional Class II Cyclase - diTS	[59]
Phoma betae Frank PS-16	BAD29971.1	aphidicolan-16β-ol synthase	Bifunctional Class II Cyclase - diTS	[60]
Phoma betae N2	BAF45924.1	fusicoccadiene synthase *PaFs*	Bifunctional Chimeric Class I Cyclase-PT - diTS	[61]
Aspergillus clavatus NRRL 1	A1C8C3.1	ophiobolin synthase *AcOS*	Bifunctional Chimeric Class I Cyclase-PT - sesterTS	[62]
Aspergillus variecolor NBRC 32302	BAU98235.1	astellifadiene synthase	Bifunctional Chimeric Class I Cyclase-PT - sesterTS	[63]
Aspergillus variecolor NBRC 32302	BAX76657.1	quiannulatene synthase	Bifunctional Chimeric Class I Cyclase-PT - sesterTS	[64]
Aspergillus variecolor NBRC 32302	BAT32888.1	variediene synthase	Bifunctional Chimeric Class I Cyclase-PT - sesterTS	[65]
Aspergillus terreus ATCC 20516	AAF13263.1	aristolochene synthase	Class I Cyclase - sesquiTS	[66]
Aspergillus fumigatus Af293	XP_747936.1	oxidosqualene cyclase/protosta17(20)Z,24-dien-3b-ol synthase	Class II Cyclase - triTS	[67]
Aspergillus flavus NRRL6541	AAT65718.1	biosynthesis of aflatrem *atmC*	IPPS PT aromatic - indolediTS	[68]
Aspergillus flavus NRRL3357	XP_002378991.1	pentalenene synthase putative	Class I Cyclase - sesquiTS	[69]
Penicillium brasilianum NBRC 6234	BBD05404.1	sesterbrasiliatriene synthase	Bifunctional Chimeric Class I Cyclase-PT - sesterTS	[70]
Penicillium verruculosus TPU1311	BBD05405.1	preasperterpenoid synthase	Bifunctional Chimeric Class I Cyclase-PT - sesterTS	[70]
Penicillium roqueforti NRRL 849	AAA33694.1	aristolochene synthase as	Class I Cyclase - sesquiTS	[71]
Penicillium paxilli PN2013	AAK11531.1	biosynthesis of paxilline (indolediterpene) *paxG*	IPPS PT aromatic - indolediTS	[72]
Botrytis cinerea T4	AAQ16575.1	presilphiperfolan-8β-ol synthase *BcBOT2*	Class I Cyclase - sesquiTS	[73]
Botrytis cinerea B05.10	XP_001550978.1	*Bcsct5* ABA biosynthesis	Class I Cyclase - sesquiTS	[74]
Cephalosporium caerulens	AAL56020.1	oxidosqualene cyclase/lanosterol synthase	Class II Cyclase - triTS	[75]
Neurospora crassa 15300	AAA19428.1	phytoene synthase-lycopene cyclase polypeptide	Class I PT GFPP synthase - Class I Cyclase - tetraTS	[76]
Neotyphodium lolii Lp19	ABF20225.1	biosynthesis of lolitrem B	IPPS PT aromatic - indolediTS	[77]
Hypoxylon sp. E7406B	AHY23922.1	1,8 cineole synthase	Class I Cyclase - monoTS	[78]
Ganoderma lucidum CGMCC 5.616	AHN91949.1	squalene synthase sqs	Class I PT - triTS	[79]
Trichoderma brevicompactum IBT40841	CAX94841.1	trichodiene synthase	Class I Cyclase - sesquiTS	[24]
Trichoderma arundinaceum IBT40837	CBX36793.1	trichodiene synthase	Class I Cyclase - sesquiTS	[21]
Trichoderma reesei QM6a	AFX82678.1	FPP synthase *erg20*	Class I PT – sesquiTS, triTS	[26]
Trichoderma virens Gv29-8	ABE60722.1	volatile sesquiterpene and monoterpene biosynthesis	Class I TS	[27]
