# Supplementary material for: Combined Comparative Genomics and Gene Expression Analyses Provide Insights into the Terpene Synthases Inventory in Trichoderma
Source: Microorganisms. 2020 Oct 18;8(10):1603. doi: 10.3390/microorganisms8101603 (PMC7603203; doi:10.3390/microorganisms8101603)
Supplement: Supplementary file 1 [file microorganisms-08-01603-s001.zip › microorganisms-945438_Supplementary/Table S3.pdf]

**Table S3. Primer sequences used for gene expression analysis (5'to 3').**

| Name | Sequence | Gene | Amplicon size |
|---|---|---|---|
| TUB-F | GCTACCTGACCTGCTGCTCTAT | *β-tubulin* | 128 bp |
| TUB-R | AGTCTGGATGTTGTTGGGGAT | | |
| TRI5s-F | TTCTGCGTCATTATGGAGGC | *tri5* | 137 bp |
| TRI5s-R | AAGGAAGCCAGGATAGTCGC | | |
| TS1-F | TCAACGCCCACTACCCAGA | *ts1* | 205 bp |
| TS1-R | ACCGTGTCGTCCCAGCAG | | |
| TS3-F | GATTTTACATCATCCCACCCC | *ts3* | 113 bp |
| TS3-R | CAGTTGGCGGAGGACTTCAG | | |
| TS4-F | ATCACAAATGGCGGCAAGA | *ts4* | 235 bp |
| TS4-R | CAGCGTGGAAGAAGAAAATAGTC | | |
| TS5-F | TGCTTAGTGTTACCGTCCTTCTG | *ts5* | 153 bp |
| TS5-R | TAATCTTCTTCTTCATCTTGGGC | | |
| TS6-F | CCTTTTCTTTGCTGTCGTGG | *ts6* | 198 bp |
| TS6-R | GCAGTTTCTCGGCTGTCATTC | | |
| TS7-F | GGTCATCTCTCCGCATTTCCC | *ts7* | 183 bp |
| TS7-R | CTCACTACTCCATTCATCGCTGTT | | |
| TS9-F | AAACCATAAACTCAGCCAACTACG | *ts9* | 198 bp |
| TS9-R | CAGTCTTGTTCCCCACCATCTC | | |
| TS11-F | CAACTCGGGCAGGCGGAC | *ts11* | 139 bp |
| TS11-R | TCGGCGATACTGTTTGAAGCA | | |
