# Supplementary material for: Combined Comparative Genomics and Gene Expression Analyses Provide Insights into the Terpene Synthases Inventory in Trichoderma
Source: Microorganisms. 2020 Oct 18;8(10):1603. doi: 10.3390/microorganisms8101603 (PMC7603203; doi:10.3390/microorganisms8101603)
Supplement: Supplementary file 1 [file microorganisms-08-01603-s001.zip › microorganisms-945438_Supplementary/Table S4.pdf]

Table S4. TS-gene content per each *Trichoderma* spp. Terpene synthase--protein groups identified by computational analysis are highlighted in different colours according to those used in Figure 1 and Figure S1, in different shadows depending on the gene amount per each species. Specific portions of the terpenoid inventory are boxed in red.

Strain	Nº TSs	*B. bassiana* ARSEF2860	*T. virens* Gv29-8	*T. pleuroticola* Tr1	*T. pleuroti* TPhu1	*T. harzianum* CBS 226.95	*T. harzianum* TR274	*T. guizhouense* NJAU 4742	*T. afroharzianum* T6776	*T. atrobrunneum* ITEM 908	*T. atroviride* IMI 206040	*T. gamsii* A5MH	*T. gamsii* T6085	*T. hamatum* GD12	*T. asperellum* TR356 v1.0	*T. asperellum* CBS 433.97	*T. arundinaceum* IBT 40837	*T. brevicompactum* IBT 40841	*T. citrinoviride* TUCIM 6016	*T. longibrachiatum* ATCC 18648	*T. parareesei* CBS 125925	*T. reesei* RUT C-30	*T. reesei* QM6a
TS-family size	387	16	20	23	23	18	18	19	20	16	18	15	16	15	21	20	20	21	18	15	16	17	18
HAD-like	7	0	0	0	0	0	0	0	0	0	1	1	1	0	2	2	0	0	0	0	0	0	0
Uncharacterized group 1	8	1	0	0	0	0	0	0	0	0	1	0	1	1	1	1	0	0	0	1	0	1	1
TRI5	7	1	0	0	0	0	0	1	0	0	0	1	1	0	1	1	1	1	0	0	0	0	0
Uncharacterized group 2	14	0	1	0	0	0	0	0	0	0	2	2	2	2	2	2	1	0	0	0	0	0	0
Longiborneol synthases	15	1	1	1	1	1	1	1	1	1	0	0	0	0	0	0	1	1	1	1	1	1	1
Presilphiperfolan-8β-ol synthases	16	0	1	2	2	1	1	1	2	0	0	0	0	0	0	0	0	1	1	1	1	1	1
Pentalenene synthases	22	0	1	1	2	1	1	1	1	1	1	1	1	1	1	1	1	1	1	1	1	1	1
Uncharacterized group 3	7	0	0	1	1	1	1	1	1	1	0	0	0	0	0	0	0	0	0	0	0	0	0
Uncharacterized group 4	58	0	5	6	4	3	3	3	3	3	3	1	1	2	2	2	3	4	3	1	2	2	2
Squalene synthases	22	1	1	1	2	1	1	1	1	1	1	1	1	1	1	1	1	1	1	1	1	1	1
GGTases 1	21	1	1	1	1	1	1	1	1	1	1	1	1	1	1	1	1	1	1	1	1	1	1
GGTases 2	21	1	1	1	1	1	1	1	1	1	1	1	1	1	1	1	1	1	1	1	1	1	1
FTases	21	1	1	1	1	1	1	1	1	1	1	1	1	1	1	1	1	1	1	1	1	1	1
Uncharacterized group 5	21	2	1	1	1	1	1	1	1	1	1	1	1	1	1	1	1	1	1	1	1	1	1
Oxidosqualene cyclases	21	1	1	1	1	1	1	1	1	1	1	1	1	1	1	1	1	1	1	1	1	1	1
Diterpene synthases	8	0	0	0	0	0	0	0	0	0	0	0	0	0	1	1	1	1	1	0	1	1	1
GGPP synthases	22	1	1	1	1	1	1	1	1	1	1	1	1	1	1	1	1	1	2	1	1	1	1
FPP synthases	31	1	1	2	2	2	2	2	2	1	1	1	1	1	1	1	3	3	1	1	1	1	1
Indole diTS	23	1	2	1	1	1	1	1	1	1	2	1	1	1	1	1	1	1	1	1	1	1	1
Chimeric-like	22	3	1	2	2	1	1	1	2	1	0	0	0	0	2	1	1	1	1	1	1	1	2
CLADE			VIRENS					HARZIANUM					VIRIDE				BREVICOMPACTUM			LONGIBRACHIATUM			
